# Supplementary figures and images for: Induction of trained immunity by influenza vaccination - impact on COVID-19
Source: PLoS Pathog. 2021 Oct 25;17(10):e1009928. doi: 10.1371/journal.ppat.1009928 (PMC8568262; doi:10.1371/journal.ppat.1009928)

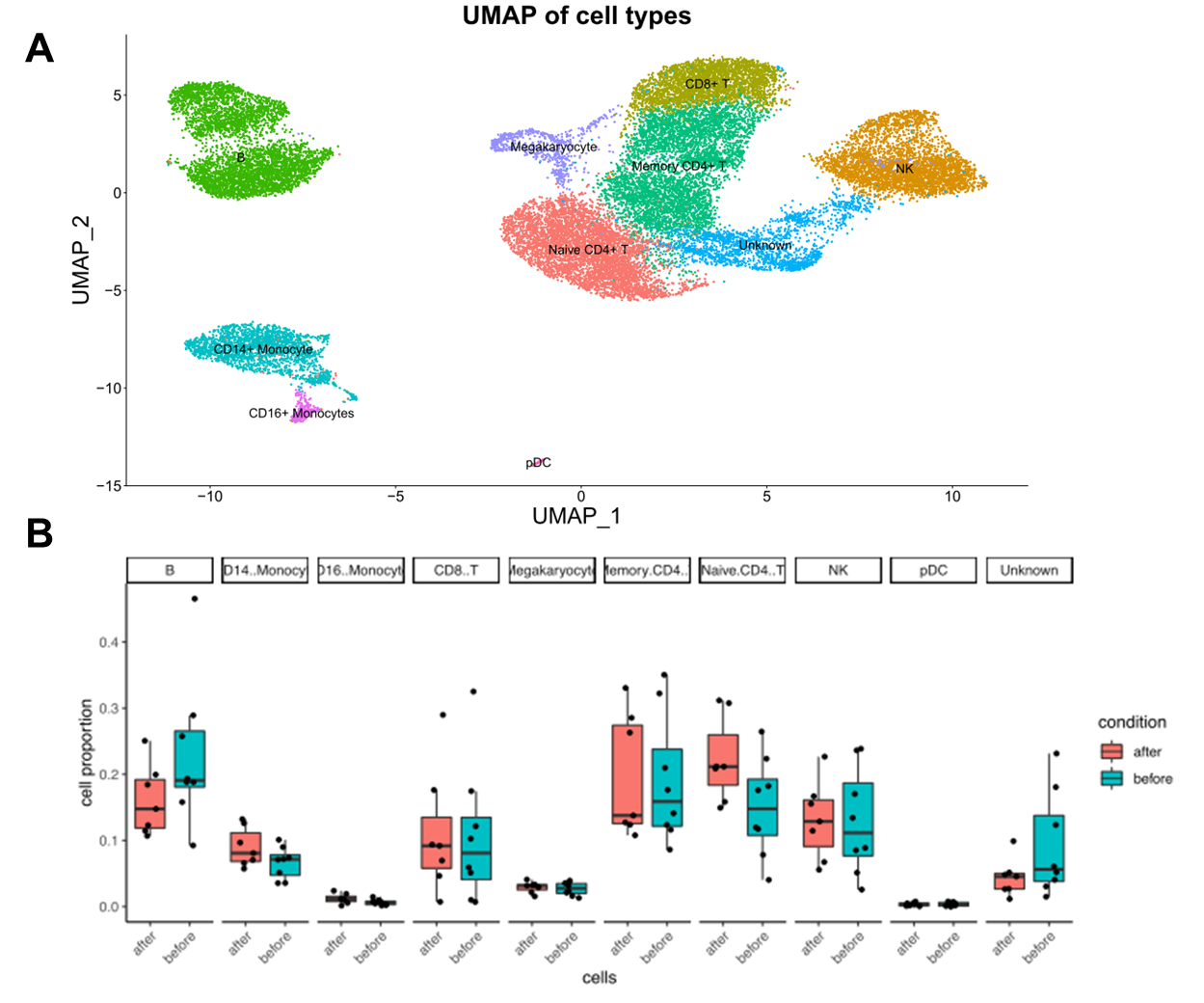

Supplement: S1 Fig — A. Two-dimensional Uniform Manifold Approximation and Projection (UMAP) embedding of 25.562 single cells. Cells are colored respective to their major cell lineages. B. Proportions of immune cell populations 1 week before and 6 weeks after the influenza vaccination. (TIF) [file ppat.1009928.s001.tif]

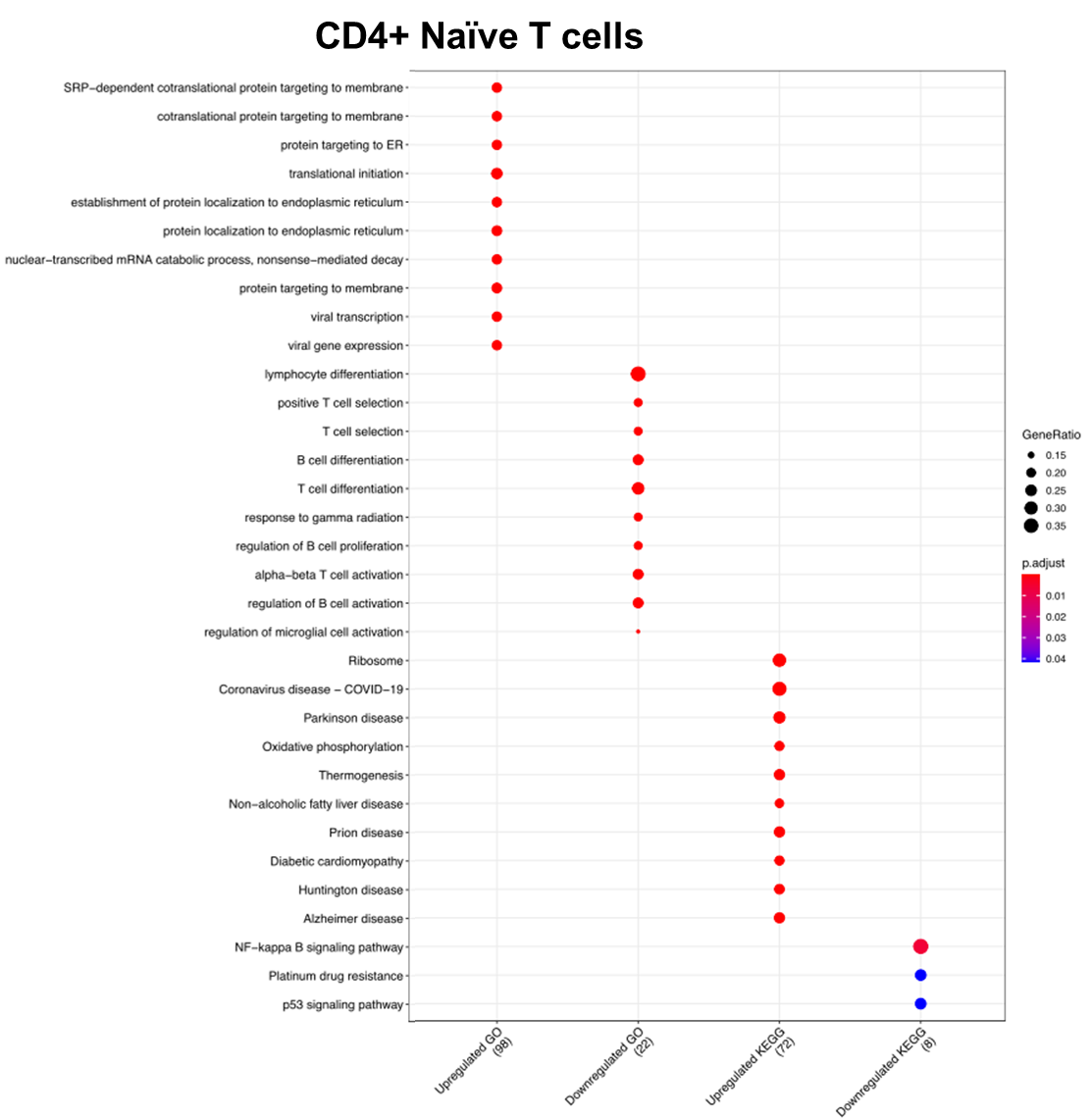

Supplement: S2 Fig — Analyses were performed with the genes whose expressions significantly change after vaccination in CD4+ naïve T cells. (TIF) [file ppat.1009928.s002.tif]

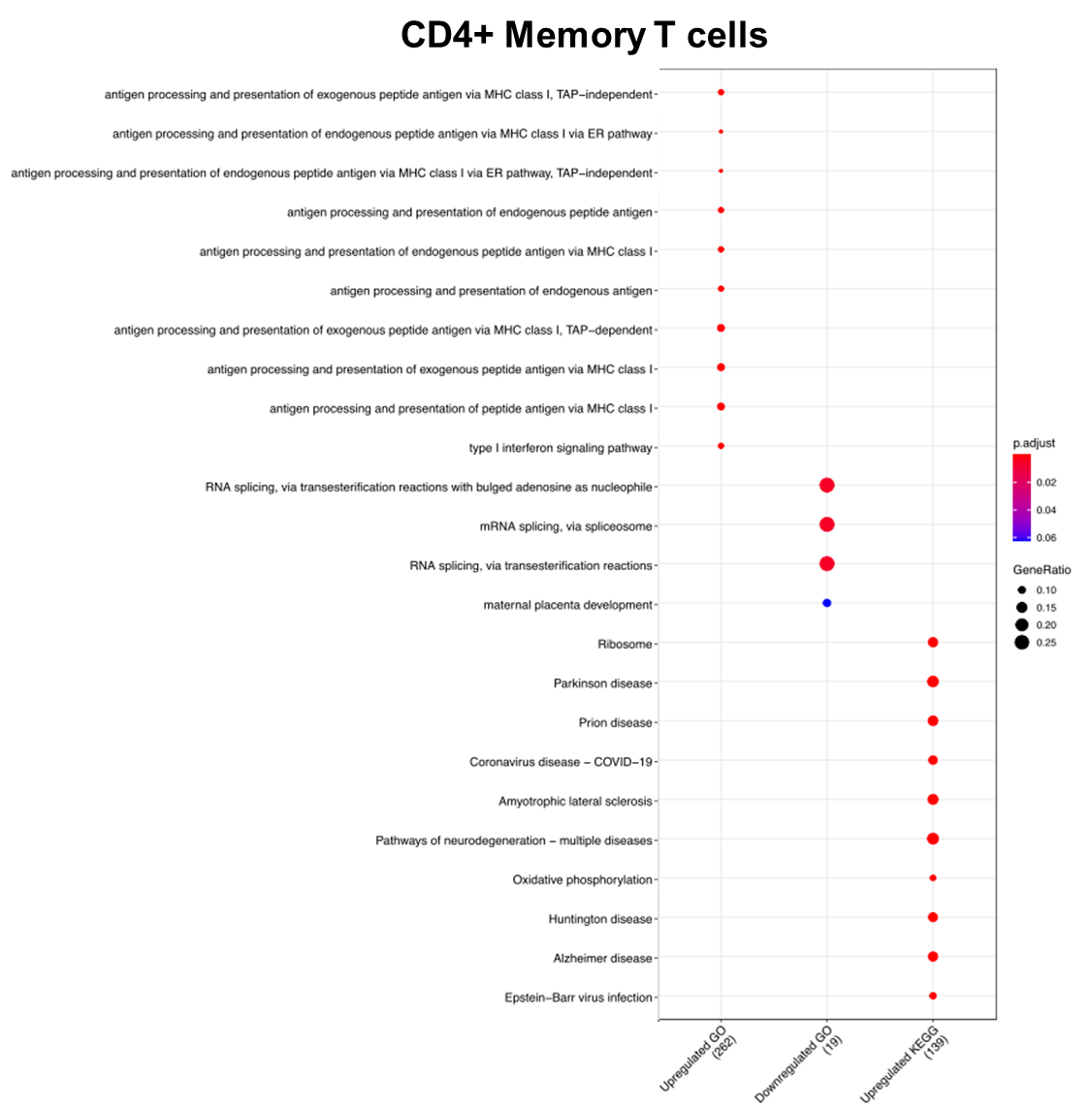

Supplement: S3 Fig — Analyses were performed with the genes whose expressions significantly change after vaccination in CD4+ memory T cells. (TIF) [file ppat.1009928.s003.tif]

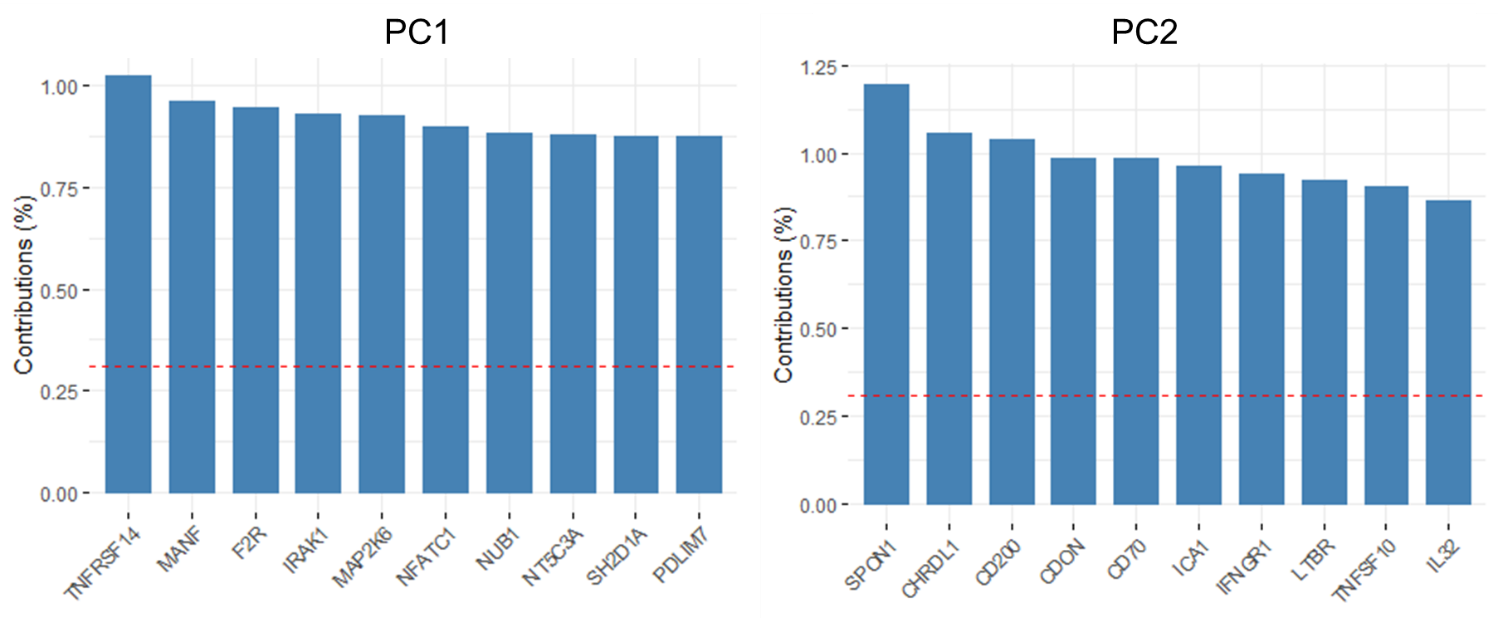

Supplement: S4 Fig — Left panel shows the proteins contributing to the variance in the first dimension (PC1), while right panel demonstrates the proteins contributing to the variance in the second dimension (PC2). (TIF) [file ppat.1009928.s004.tif]

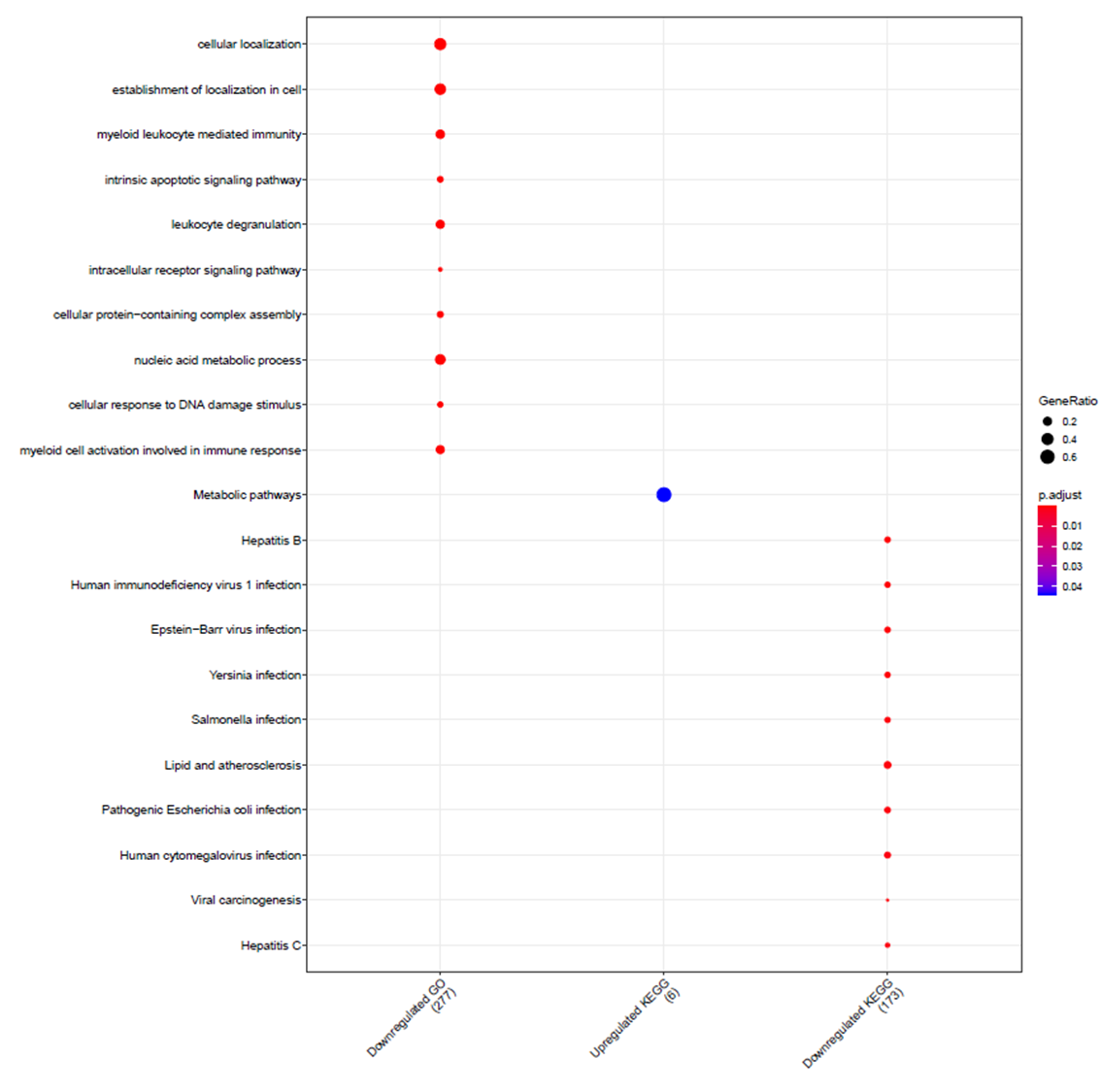

Supplement: S5 Fig — The complete Olink panel of 1472 proteins were used as the background in the enrichment analysis. (TIF) [file ppat.1009928.s005.tif]

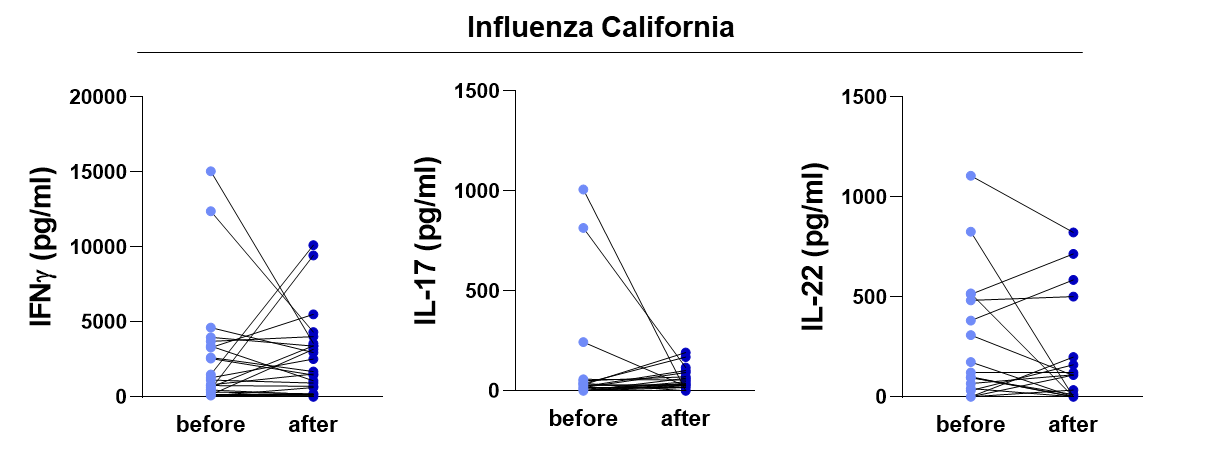

Supplement: S6 Fig — PBMCs were stimulated with heat-killed Influenza H1N1 (California strain) for 7 days. IFNγ, IL-17 and IL-22 responses were quantified. Wilcoxon signed-rank test revealed no significant differences in cytokine production between before and after influenza vaccination. (TIF) [file ppat.1009928.s006.tif]
