# Supplementary material for: Induction of trained immunity by influenza vaccination - impact on COVID-19
Source: PLoS Pathog. 2021 Oct 25;17(10):e1009928. doi: 10.1371/journal.ppat.1009928 (PMC8568262; doi:10.1371/journal.ppat.1009928)
Supplement: S1 Table — (DOCX) [file ppat.1009928.s007.docx]

**S1 Table. Studies on the association between influenza vaccination and COVID-19 related outcomes.**

| Author, year | Design | Methods | Main results |
| --- | --- | --- | --- |
| Hernandez et al., 2020 [1] | Ecological study | COVID-19 mortality in the elderly (> 65 years) and influenza VCR were analyzed across 21 regions in Italy. No correction for confounding was performed.  Data up to May 2020 were used. | Negative correlation between influenza vaccination and COVID-19 attributable mortality. (r = −.5874, n = 21, *P* = .0051) |
| Amato et al., 2020 [2] | Ecological study | Influenza VCR and SARS-CoV-2 seroprevalence, hospitalization rates, ICU admissions, and COVID-19 related mortality in the elderly (>65 years) in Italy were assessed. Correction for confounders was performed for several comorbidities, economic and environmental variables. Data were collected from March until June 2020. | Negative correlation between influenza vaccination and SARS-CoV-2 seroprevalence, hospitalization rates, ICU admission as well as COVID-19 attributable mortality.  (R^2^ of 0.88, 0.82, 0.70 and 0.78 respectively) |
| Zanettini et al.,  2020 [3] | Ecological  study | COVID-19 mortality in the elderly (> 65 years) and influenza VCR were analyzed across the USA. Correction for confounding was performed for several socio-economic-, demographic-, healthcare-, race- and comorbidity- related variables.  Data from January 2020 until June 2020 were used. | Negative association between influenza vaccination and COVID-19 attributable mortality, where every 10% increase in influenza VCR, causes a 28% decrease in COVID-19 related death.  (MRR = 0.72; 95%CI: 0.58-0.89) |
| Arokiaraj, 2020 [4] | Ecological study | COVID-19 morbidity and mortality in the elderly (> 65 years) and influenza VCR were analyzed across OECD countries. | Negative correlations between influenza vaccination and the COVID-19 epidemiological parameters. However, it lacks proper statistical analysis. |
| Lisewski et al., 2020 [5] | Ecological study | COVID-19 incidence in the elderly (> 65 years) and influenza VCR were analyzed across 29 OECD countries. | Positive correlation between influenza vaccination and COVID-19 attack rates.  (r = 0.58; 95%CI: 0.27 to 0.78; *P*=.001) |
| EBMPHET consortium, 2020 [6] | Ecological study | COVID-19 mortality in the elderly (> 65 years) and influenza VCR were analyzed across Europe and the USA.  Data up to May 2020 were used.  No correction for confounding was performed. | Positive correlation between influenza vaccination and COVID-19 incidence in Europe (r = 0.66 ± 0.13, P = .000017) and the USA (r = 0.50 ± 0.14, *P* <.05). Positive correlation between influenza vaccination and COVID-19 attributable mortality in Europe (r = 0.68 ± 0.13, p = 0.000006) and the USA (r = 0.50 ± 0.14, *P* <.05). |
| Fink et al., 2020 [7] | Cross-sectional  study | Associations between COVID-19 related mortality, intensive care treatment, need for invasive respiratory support and influenza VCR were analyzed in 92.664 clinically and molecularly confirmed COVID-19 cases in Brazil. Correction for confounding was performed for several comorbidities, sociodemographic factors, and healthcare facilities. | Positive correlation between influenza vaccination and COVID-19 attributed mortality (17% lower odds, 95%CI [0.75,0.89]). Positive correlation between influenza vaccination and need for ICU treatment (8% lower odds, 95%CI [0.86,0.99]). Positive correlation between influenza vaccination and need for invasive respiratory support (18% lower odds, 95%CI [0.74,0.88]) |
| Jehi et al.,  2020 [8] | Ecological  study | An individualized risk prediction model was created using a registry of 11,672 patients tested for COVID-19 in Cleveland Clinic. The multivariable logistic model was constructed based on demographics, symptoms, comorbidities, laboratory variables, medications, immunization history, and travel history. Validation was performed in a temporally and geographically distinct cohort of 2295 individuals. | Reduced positive test risk in people who had an influenza vaccine. 93.9% influenza vaccination rate in the COVID-negative group, in contrast to 6.1% in the COVID-positive group (P < .001) in the development cohort. The rates were 91.6% and 8.4% (P < .011), respectively, in the validation cohort. |
| Yang et al.,  2021 [9] | Ecological  study | Associations between influenza vaccination and COVID-19 related hospitalizations and ICU admissions were investigated in Florida. Data were controlled for age, gender, race, and comorbidities such as hypertension, diabetes, and coronary artery disease. | 2.44 greater odds of hospitalization (95% CI, 1.68, 3.61) and 3.29 greater odds of ICU admission (95% CI, 1.18, 13.77) in COVID-19 patients who did not receive an influenza vaccination in the last year. |
| ICU, intensive care unit; MRR, mortality rate ratio; OECD, Organization for Economic Cooperation and Development; VCR, vaccination coverage rate. | | | |

**References**

1. Marin-Hernandez D, Schwartz RE, and Nixon DF. Epidemiological evidence for association between higher influenza vaccine uptake in the elderly and lower COVID-19 deaths in Italy. J Med Virol. 2021;93(1):64-5.

2. Amato M, Werba JP, Frigerio B, Coggi D, Sansaro D, Ravani A, et al. Relationship between Influenza Vaccination Coverage Rate and COVID-19 Outbreak: An Italian Ecological Study. Vaccines (Basel). 2020;8(3).

3. Zanettini C, Omar M, Dinalankara W, Imada EL, Colantuoni E, Parmigiani G, et al. Influenza Vaccination and COVID-19 Mortality in the USA: An Ecological Study. Vaccines (Basel). 2021;9(5).

4. Arokiaraj MC. Correlation of influenza vaccination and influenza incidence on COVID-19 severity. 2020. Available at SSRN: <https://ssrn.com/abstract=3572814> [doi:10.2139/ssrn.3572814](https://dx.doi.org/10.2139/ssrn.3572814)

5. Lisewski AM. Association between influenza vaccination rates and SARS-CoV-2 outbreak infection rates in OECD countries. 2020. Available at SSRN: <https://ssrn.com/abstract=3558270> [doi:10.2139/ssrn.3558270](https://dx.doi.org/10.2139/ssrn.3558270)

6. EBMPHET Consortium. COVID-19 severity in Europe and the USA: Could the seasonal influenza vaccination play a role? 2020. Available at SSRN: <https://ssrn.com/abstract=3621446> [doi:10.2139/ssrn.3621446](https://dx.doi.org/10.2139/ssrn.3621446)

7. Fink G, Orlova-Fink N, Schindler T, Grisi S, Ferrer APS, Daubenberger C, et al. Inactivated trivalent influenza vaccination is associated with lower mortality among patients with COVID-19 in Brazil. BMJ Evid Based Med. 2021, 26.4: 192-193.

8. Jehi L, Ji X, Milinovich A, Erzurum S, Rubin BP, Gordon S, et al. Individualizing Risk Prediction for Positive Coronavirus Disease 2019 Testing: Results From 11,672 Patients. Chest. 2020;158(4):1364-75.

9. Yang MJ, Rooks BJ, Le TT, Santiago IO, 3rd, Diamond J, Dorsey NL, et al. Influenza Vaccination and Hospitalizations Among COVID-19 Infected Adults. J Am Board Fam Med. 2021;34(Suppl):S179-S82.
